# Pneumonia in Bhutanese children: what we know, and what we need to know

**DOI:** 10.1186/s41479-019-0065-x

**Published:** 2020-01-25

**Authors:** Sophie Jullien, Dinesh Pradhan, Quique Bassat

**Affiliations:** 1 ISGlobal, Hospital Clínic, Universitat de Barcelona, Barcelona, Spain; 2 Jigme Dorji Wangchuck National Referral Hospital, Thimphu, Bhutan; 3 Khesar Gyalpo University of Medical Sciences of Bhutan (KGUMSB), Thimphu, Bhutan; 4 ICREA, Pg. Lluís Companys 23, 08010 Barcelona, Spain; 5 Paediatric Infectious Diseases Unit, Paediatrics Department, Hospital Sant Joan de Déu (University of Barcelona), Barcelona, Spain; 6 Centro de Investigação em Saúde de Manhiça (CISM), Maputo, Mozambique; 7 CIBER of Epidemiology and Public Health, CIBERESP, Madrid, Spain

**Keywords:** Pneumonia, Respiratory tract infections, Bhutan, Viruses, Infant, Child preschool, Epidemiology, Vaccines

## Abstract

**Background:**

Pneumonia is the single largest cause of death in under-five children worldwide. We conducted a systematic review to identify the knowledge gaps around childhood pneumonia in Bhutan.

**Methods:**

We searched PubMed, ScienceDirect and Google scholar from conception to 3rd December 2018, World Health Organization, UNICEF, Bhutan’s Ministry of Health and other local databases for relevant reports. We included any report describing pneumonia in Bhutanese children with regards to the burden of the disease, aetiology, related risk factors, clinical and prognostic characteristics, surveillance systems and national preventive strategies. Two review authors identified the records. We summarized the findings narratively.

**Results:**

We included 44 records. Although with notable decreasing trends, pneumonia is still accountable for a high burden and mortality rate in Bhutanese children. The national surveillance system focuses mainly on influenza identification but has recently introduced other viral aetiology to monitor. We found very scarce or no data with regard to the bacterial aetiology, related risk factors and clinico-radiological and prognostic characteristics.

**Conclusion:**

There is a dearth of data regarding the epidemiological, microbiological, clinical and radiological characteristics of pneumonia in children in Bhutan, leading to challenges while implementing evidence-based management and effective national preventive strategies.

## Background

In 2015, pneumonia was ranked as the single biggest killer of post-neonatal children worldwide. With an estimated 15·5% attributable fraction of all deaths in children under 5 years of age, pneumonia is believed to be responsible for the deaths of around 900,000 children every year [1, 2]. The main burden remains disproportionately concentrated in low- and middle-income countries (LMIC) in Southeast Asia and sub-Saharan Africa, where pneumonia is one of the most frequent triggers of health facility consultation, and one of the most common causes of hospitalization, representing a huge load for the overburdened and fragile health care systems [3].

The world has achieved considerable progress in reducing child mortality in the last two decades [4], and reductions in pneumonia-attributable mortality are partly responsible for such a massive public health achievement [1]. Various interventions have shown to be effective to prevent and treat childhood pneumonia, including exclusive breastfeeding for 6 months, prevention of childhood malnutrition, childhood immunization, reduction of household air pollution, hand washing, and use of simple and standardized guidelines [5–7]. Although substantial progress has been achieved, the toll of preventable deaths related to pneumonia remains intolerably heavy. Better implementation of the strategies shown to be effective to prevent and treat childhood pneumonia seems crucial to further reduce the burden of pneumonia. Once implemented, adequate national surveillance systems are required to evaluate their progress towards the reduction of the disease burden. To this end, it is imperative to collect reliable local data to determine the burden of the disease, the main pathogens involved, risk factors contributing to the disease, and to describe epidemiological trends. However, reliable local data are often scarce or entirely missing in LMIC, where the main burden of pneumonia remains concentrated, leading to challenges when it comes to evaluating the preventive strategies implemented [8].

Bhutan is a small country (38,394 km^2^) locked in the Himalayas between India and China [9]. Elevation rises from around 100 m to more than 7500, with the capital, Thimphu, standing at 2334 m [10]. It is currently classified as a lower-middle income country [11]. The population was estimated at 779,666 in 2017 and life expectancy at birth was 70·6 years in 2016 [12, 13]. Two thirds of the population live in rural areas [14]. Essential health services in both modern and traditional medicines are free for Bhutanese citizens, as guaranteed by the Constitution, based on a primary health care approach [15]. Health services are offered across the country through secondary and tertiary level hospitals, basic health units, subposts and outreach clinics [12].

The WHO-recommended Integrated Management of Childhood Illnesses (IMCI) strategy was adopted and implemented in Bhutan in 2000, as a strategy to address the major causes of deaths in under-five children. Health workers were trained across the country and the coverage of IMCI implementation reached 100% in all the districts of the country by 2011 [16]. The strategy promotes the early recognition of different syndromes (including pneumonia) in under-five children, as well as a simple and algorithm-based systematic approach for their management. Despite these efforts, acute respiratory infections (ARI) seem to remain a major public health challenge in Bhutan, whereby they were estimated to represent the fifth cause of mortality for the whole population, and to cause 15% of deaths in under-five children [12, 17]. The conjugate *Haemophilus influenzae* type b (Hib) vaccine is routinely administered since 2011 and the pneumococcal conjugate vaccine was recently introduced in the childhood immunization schedule (January 2019) [18, 19]. Reduction of risk factors, immunisation, and case management are the main approaches to disease control [3].

Summarizing the available data on the epidemiology, aetiology and clinical characteristics of pneumonia among Bhutanese children would help to better understand and characterize the impact of this major killer and would allow the identification of knowledge gaps to improve local tailored programmes to reduce the burden and mortality associated with childhood pneumonia in Bhutan.

## Objectives

We conducted this systematic review in order to identify the knowledge gaps around ARI in children in Bhutan. We aimed to collect any available data with regards to the burden of the disease, the aetiology, clinico-radiological characteristics, the associated health determinants and trends over the last years, as well as the current surveillance systems and national preventive strategies.

## Methods

We followed the protocol established for this review (Additional file 1: Appendix 1).

### Selection criteria

We sought to include any study or primary report in which participants were children under 5 years of age (or all ages when disaggregated data were not available) in Bhutan that would report data on ARI or pneumonia, as defined by the study authors, regarding any of the following:

- burden of the disease such as incidence, prevalence, morbidity, mortality, or number of visits in health facilities;
- aetiology;
- related risk factors;
- clinical and prognostic characteristics;
- management;
- existing surveillance systems;
- national preventive strategies related to ARI or pneumonia.

We excluded documents when duplicated.

### Definitions

The clinical case definition of pneumonia has been changing over the last few decades [20, 21]. As of today, there is no optimal gold standard definition available [22]. Lower respiratory infections (LRI) is a broad term that includes pneumonia and bronchiolitis, as defined in the Global Burden of Diseases, Injuries, and Risk Factors Study (GBD) [6]. This is the terminology we strived to use in this review. However, we will report data as defined by the study authors. Thereby, ARI is often used and may imply here a broader concept including both upper and lower respiratory infections.

### Search methods for identification of studies

We attempted to identify all relevant reports regardless of language or publication status (published, unpublished, and in press).

#### Electronic searches

We conducted an electronic search of the following databases, using the search terms and strategy described in Additional file 1: Appendix 2. PubMed (1942 to 3 December 2018), ScienceDirect (1944 to 3 December 2018) and Google Scholar (up to December 2018). We manually searched all the issues of the Bhutan Health Journal for relevant publications (conception to December 2018). We searched databases of the WHO, UNICEF, Ministry of Health in Bhutan, Royal Centre for Disease Control (RCDC), National Statistics Bureau, Jigme Dorji Wangchuck National Referral Hospital (JDWNRH) and Khesar Gyalpo University of Medical Sciences of Bhutan (KGUMSB) websites for relevant documents.

#### Other sources

We manually searched the reference lists of all documents identified by the above methods that met our eligibility criteria for other potentially relevant documents. We looked for unpublished theses or other unpublished documents at the libraries of the Faculty of Nursing and Public Health and Faculty of Postgraduate Medicine at KGUMSB in Thimphu, Bhutan. We contacted researchers and key stakeholders at the Ministry of Health, at the Microbiology Department at JDWNRH, and at RCDC in Thimphu, to identify additional potentially relevant unpublished data.

### Data collection and analysis

#### Selection of studies

Two review authors (SJ and DP) independently screened the titles and abstracts of the reports identified by the electronic searches and identified in the reference lists of selected documents or in the libraries cited above, to identify eligibility criteria. Duplicate reports were removed. We retrieved the full-text articles of the records that were identified as potentially eligible. SJ and DP independently assessed the full-text articles for eligibility, using the predefined inclusion and exclusion criteria, and resolved any disagreements by discussion.

#### Data extraction, data management and data synthesis

SJ extracted data from the included records. DP checked all extracted data to identify any possible errors. Data describing study setting, population, methods and outcomes as well as pneumonia definitions used by the study authors were extracted. From other documents and data identified from the screened websites, we extracted additional relevant data which were classified under selected themes according to our outcomes of interest. When available, we collected data for each outcome on population characteristics, setting and methodology used. We synthesized the findings narratively with the support of graphs and tables, under different themes. We reorganised the themes based on the amount of information gathered for each of them.

## Results

### Results of the search

**Fig. 1:**
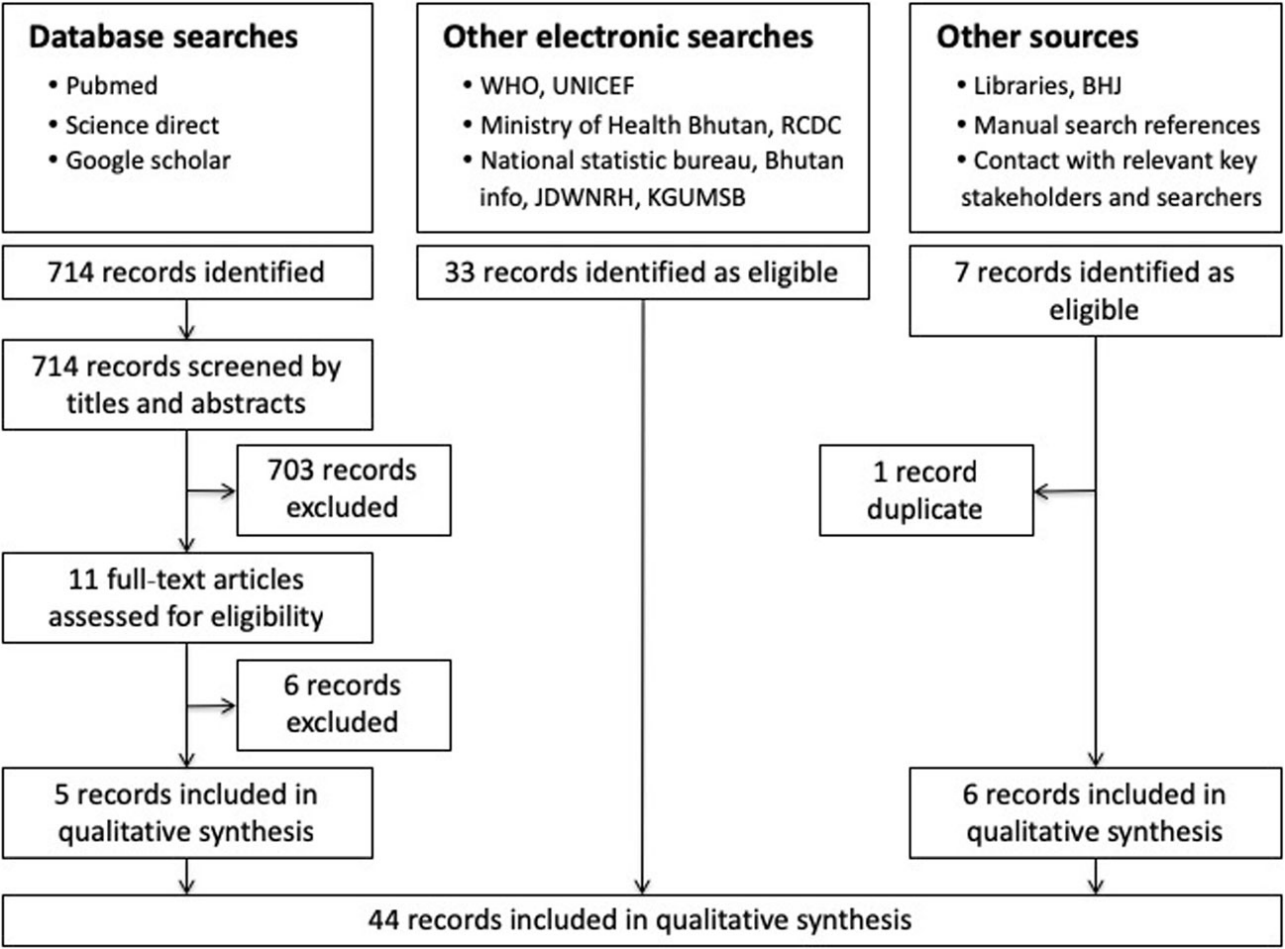
Flow diagram illustrating the selection of records

The database searches conducted up to 3rd December 2018 returned 714 records. The screening of titles and abstracts revealed 11 relevant records, for which the full-text article was retrieved. Five studies met our eligibility criteria and were included in the review. We identified 40 additional reports related to the topic through other electronic search and sources, out of which we excluded one due to duplication. Overall, we included 44 documents for qualitative synthesis (Fig. 1). Included and excluded records with reasons for exclusion are listed in Additional file 1: Appendix 3.

We included 10 studies and 34 reports. All of them are published except one unpublished report and one medical thesis Tshering T: Incidence, risk factors, and outcome of ventilator-associated pneumonia in pediatric intensive care unit Jigme Dorji Wangchuk National Referral Hospital. A prospective hospital-based study, (unpublished observations) (see Additional file 1: Appendix 3). The medical thesis focuses on ventilator-associated pneumonia (VAP) in children admitted at the national referral hospital (JDWNRH), while all the other studies describe community acquired infections. Eight studies focused on Bhutan, while two manuscripts presented data on LRI in 195 countries including Bhutan [6, 23]. Nine out of the 10 studies were published in the last 8 years; the remaining one was published in 1995 [24]. Only two studies [24, 25] focused on children, all other studies included all age groups.

### Surveillance systems for respiratory infections in Bhutan

No systematic national notifiable disease surveillance system existed in Bhutan a decade ago, albeit a few notifiable diseases were reported nationally [26]. Over the last 10 years, the RCDC initiated an influenza surveillance system among outpatients and hospitalized patients, comprising Influenza-Like Illness (ILI) and Severe Acute Respiratory Infection (SARI). This clinical and laboratory-based surveillance is carried out at 11 hospitals that were selected as sentinel sites. Each sentinel site collects random samples from few ILI cases every week, and samples from every SARI case [27]. This system works as epidemiological and virological surveillance, with the aim of monitoring the burden and trends in respiratory diseases, as well as monitoring the epidemiology of influenza viruses and other respiratory pathogens [28, 29]. Data are reported on a weekly basis and feedbacks are shared on the RCDC website every week and in quarterly bulletins [15, 28, 30]. Evaluation of the SARI surveillance platform by Thapa et al. in 2015 and 2016 identified significant underreporting of SARI cases, consequently questioning the national data ensuing from this surveillance system [31].

### Burden and seasonality patterns of acute respiratory infections in Bhutan

We summarized the main findings on the burden of ARIs in Bhutan in Additional file 1: Appendix 4, Table 4A.

The incidence of pneumonia in Bhutan has exhibited a decreasing trend in the last 10 years, from 1479 cases per 10,000 children under 5 years of age in 2008 to 809 in 2017 [12, 32]. Pneumonia cases, number of outpatient visits attributed to pneumonia in hospitals and in basic health units, and number of inpatients attributed to pneumonia in under-five children have also decreased in the last 10 years in term of both absolute and relative numbers (Additional file 1: Appendix 4, Table 4B).

According to the quarterly bulletins published by the RCDC, the national surveillance system reported a total of 172,833 cases of respiratory illness in 2018 [30] Additional file 1: Appendix 4, Table 4C.

One study aimed to estimate influenza-associated respiratory hospitalization rates in Bhutan [31]. There were 11,782 hospital discharges coded with respiratory diagnosis in 2015 across Bhutan, and 13,697 in 2016. Six district hospitals identified as sentinel sites that were included in their analysis reported 3138 respiratory hospitalizations, of which 45% were among under-five children. The highest influenza-associated hospitalization rates were found among children under five: 182 (95% CI: 153 to 210) and 532 (95% CI: 473 to 591) per 100,000 persons in 2015 and 2016, respectively.

Looking at the most updated available data, the influenza sentinel surveillance reported 1214 cases of ILI per 10,000 hospital visits and 29 cases of SARI per 100 hospital admissions in 2018 [30] (Additional file 1: Appendix 4, Table 4D).

Finally, one record looked at VAP in children admitted in the paediatric intensive care unit in 2017. Out of the 92 included patients, 13 were diagnosed as VAP, resulting in VAP incidence of 14·1% and in VAP incidence density of 44·9/1000 ventilator days.

**Fig. 2:**
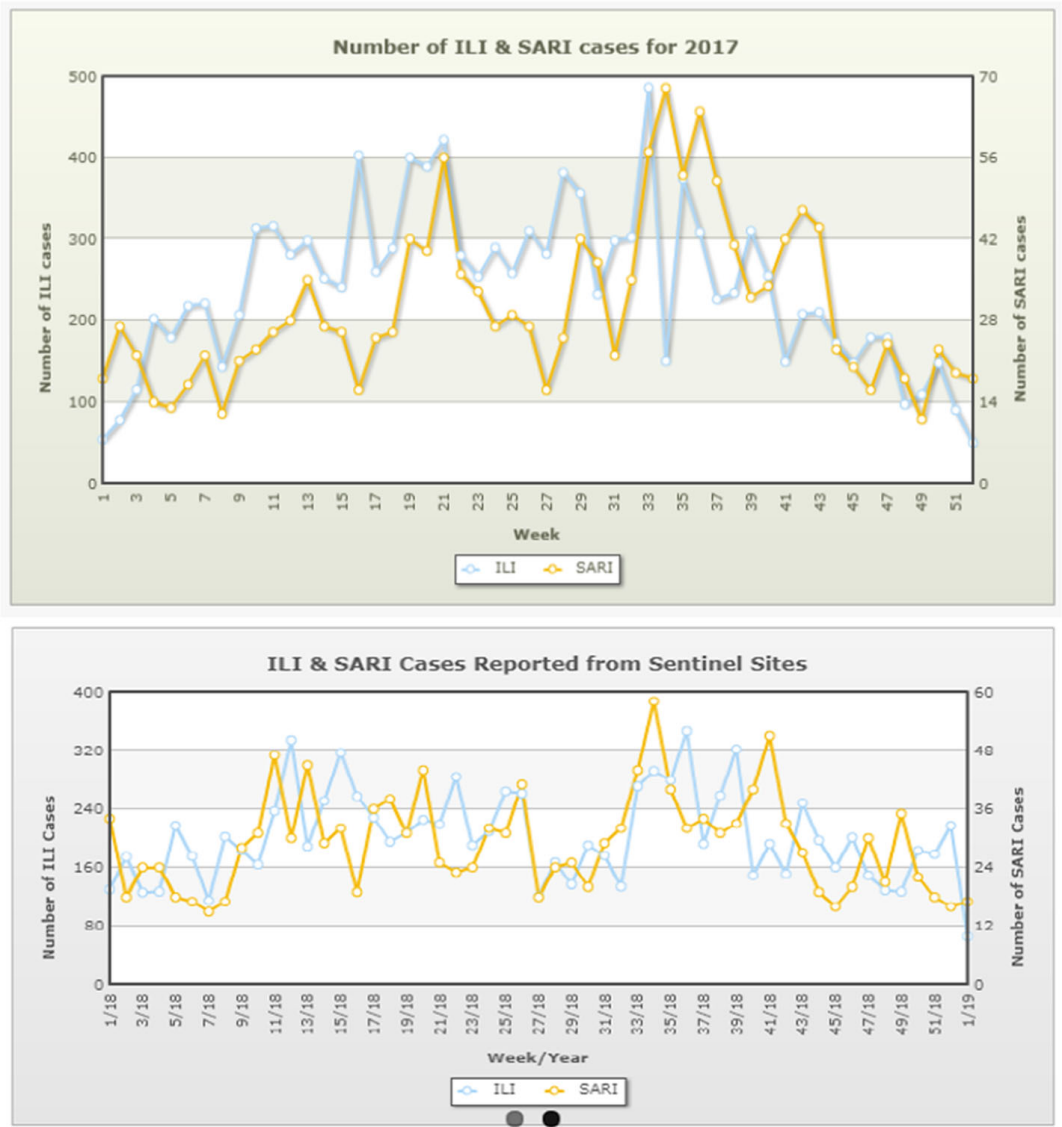
Number of ILI and SARI cases in 2017 and 2018. Reproduction permitted. Source: RCDC Fluview weekly reports [30]

Regarding the seasonality pattern of ARI, Fig. 2 shows the number of ILI and SARI cases reported weekly in the last 2 years. There are two main peaks each year, around the weeks 20 and 35 in 2017 and around the weeks 11 and 34 in 2018.

### Mortality related to acute respiratory infections and pneumonia in Bhutanese children

We summarized the main findings on the mortality related to ARIs in Additional file 1: Appendix 5, Table 5A.

Since 2015, WHO ranked pneumonia as the leading individual cause of under-five mortality worldwide, and Bhutan is not an exception. In 2016 ARI was estimated to be responsible for 15 and 27% of under-five and post-neonatal deaths, respectively. Table 1 summarizes changes in pneumonia-attributable mortality between the years 2000 and 2016 globally and for Bhutan [2].

**Table 1:** Estimates of child deaths due to acute respiratory infections. Source: UNICEF [2]

		Total under 5 deaths	Total post-neonatal death	Under 5 deaths due to ARI			Post-neonatal death due to ARI^a^		
				Absolute number	Rate (per 1000 live births)	%	Absolute number	Rate (per 1000 live births)	%
Global	2000	10,050,919	6,047,778	1,774,025	14	18	1,466,299	11	24
	2016	5,649,439	3,036,996	878,829	6	16	719,413	5	24
Bhutan	2000	1280	768	278	17	22	242	15	32
	2016	468	206	69	5	15	56	4	27

*Abbreviations*: *ARI* acute respiratory infections

^a^Calculated as (Under5 deaths due to ARI – neonatal deaths due to ARI)

The GBD study estimated an increasing LRI mortality rate from 51 to 80 per 100,000 under-five children in 2015 to 100 to 249 in 2016 [6, 23].

Focusing on data collected under the term “pneumonia”, this disease was ranked as the fifth main cause of mortality among the Bhutanese population in 2017 [12]. The estimates of deaths for different major causes in Bhutanese children between one and 59 months of age showed a decline from 2000 to 2010 in the number of deaths attributed to pneumonia (See Additional file 1: Appendix 5, Table 5B). However, pneumonia remains the single biggest cause of post-neonatal child deaths, causing 27·8% of the overall number of deaths in 2010 [25]. A hospital-based study conducted between 2009 and 2011 collected causes of under-five mortality in Bhutan. Authors reported that pneumonia accounted for 45% of deaths in children from one to 59 months, but underlined that their analysis provided data on child mortality happening in health facilities, which is likely to overestimate the true value [25]. Finally, the scarce data available on VAP in Bhutanese children describe five deaths (38·5%) out of the 13 cases of VAP included in the study.

### Aetiology of ARI

We found scarce data regarding the aetiology of ARI in Bhutan, most of which focused on influenza virus, mainly collected through the national surveillance system. We identified one paper investigating *Streptococcus pneumoniae*, and one thesis reporting on bacterial isolates for VAP.

Wangchuk et al. summarized epidemiological data on influenza from November 2008 to 2011, which includes the pandemic influenza A(H1N1)pdm09 period [33]. Influenza strains circulating prior to the pandemic period included A/H1 and A/H3, with A (H1N1)pdm09 remaining a dominant strain for almost 1 year after the pandemic period, when it was replaced by A/H3. Influenza B was present throughout and after the pandemic period. During the pandemic period, 2149 samples were collected with results shown in Additional file 1: Appendix 6, Table 6A.

The RCDC publishes “Fluview”, a weekly summary of the ILI and SARI surveillance [30]. Every week, each of the 11 hospitals identified as sentinel sites collect samples from between four and six cases of ILI, and from each cases of SARI. The respiratory samples collected at the sentinel sites are firstly tested by Real Time Polymerase Chain Reaction for Flu A/H1, Flu A/H3, Flu A/H5N1, Flu A/H7N9, Flu A/unsub and Flu B. For those samples negative to any type of influenza, there is a further attempt to identify respiratory syncytial virus (RSV) and human metapneumovirus (HMPV). Recently, identification of further viruses also includes adenovirus and parainfluenza viruses 1, 2 and 3, reported in the last two quarterly bulletins (from mid 2018) (personal communication). In 2018, RSV was identified in a considerable number of cases, while HMPV, adenovirus and parainfluenza viruses were isolated in few cases only [30].

Tshokey et al. presented the serotypes and antibiotic susceptibility of *S. pneumoniae* isolates collected at the JDWNRH in Thimphu in 2014 and 2015 [34]. Fifteen different serotypes were identified out of 21 confirmed pneumococcal isolates, collected from several sources (five were from blood sample and two from sputum) (Additional file 1: Appendix 6, Table 6B). Authors also looked at the serotype coverage from the available pneumococcal vaccines and calculated that the 7-valent and 10-valent vaccines would cover 26·7% and 40·0% of the identified serotypes respectively, while the 13-valent and 23-valent would cover 53·3% of them.

Unpublished data provided by the Department of Microbiology from the same hospital, JDWNRH in Thimphu, described the number of pneumococcal isolates and their antibiotic susceptibility in 2016. Only nine isolates were identified from blood samples, and 12 from respiratory samples, all of them fully susceptible to penicillin (Additional file 1: Appendix 6, Table 6C).

Finally, there were 18 bacterial isolates from endotracheal aspirates of 13 children with VAP reported in the medical thesis. *Klebsiella* spp. [8] and *Escherichia coli* [4] were the most common pathogens identified, followed by *Acinetobacter* spp. [2], *Enterococcus* [2], *Staphilococcus aureus* [2] and *Pseudomonas* spp. [1]. Antibiotic susceptibility was not reported for these bacterial isolates.

### Risk factors for ARI

One study published in 1995 described risk factors of ARI [24]. This prospective cohort study of 113 children born in 1990 found a statistically significant reduction in the incidence of both diarrhoea and respiratory tract infections associated with breastfeeding. Tashi and colleagues also looked at risk factors for prediction of VAP among a limited number of patients. Amongst the medical co-morbidities, disorders of the central nervous system, trauma and sepsis were statistically significant in the development of VAP.

The GBD study calculated modelling estimates in under-five LRI mortality attributable to change in risk factors between 2000 and 2016. In Bhutan, it was estimated that changes related to childhood wasting, stunting and overweight have reduced LRI-related mortality by 9·41%, 3·56% and 4·45% respectively. Similarly, changes related to handwashing have reduced LRI-related mortality by 0·38%, breastfeeding by 1·29%, second-hand smoking by 0·23%, household air pollution by 7·52%, adequate antibiotic treatment by 3·01%, zinc deficiency by 0·29% and Hib vaccination by 6·44%. The only assessed risk factor that was attributed to an increase in LRI-related mortality was ambient particulate matter pollution, with a 7·33% increase [6].

### Clinico-radiological characteristics, prognosis and management of ARI

We did not find any study describing the clinical or radiological characteristics, prognosis and management of Bhutanese children suffering from community-acquired pneumonia or ARI. The only data available suggest that in 2010, 74·2% of children under five with suspected pneumonia were taken to an appropriate healthcare provider, and that treatment with antibiotics was given in 48·7% of the cases with suspected pneumonia [35]. Tashi and colleagues presented 13 children with VAP. Most of them had important co-morbidities, including sepsis [7], congenital heart disease [6] and central nervous system infection [5] as primary diagnosis. The median duration of stay in the intensive care unit was 28 days while it was 5 days for children without VAP.

### National preventive strategies

Activities for ARI control were initiated in 1987. The ARI Programme was instituted in the erstwhile Department of Health in 1992 and activities were intensified in 1993 with the primary intention of reducing under-five deaths due to pneumonia. WHO Standard Case Management Protocol for ARI was introduced in 1994, while IMCI was implemented in 2000 [12].

In the report dedicated to the identification of under-five deaths in health facilities in Bhutan, the Ministry of Health supported by UNICEF recognised the need of expanding the role of village health workers to provide antibiotics for pneumonia in order to help reduce child mortality attributable to sepsis and pneumonia [25]. Models were developed to estimate the impact of different interventions for reducing the under-five mortality rate, and so as to identify priority child health interventions that would result in maximum impact. It was estimated that oral antibiotics for the management of pneumonia would contribute to prevent 16% of additional deaths in under-five children [25].

A health economics analysis was conducted to determine the cost-utility of 10- and 13-valent pneumococcal conjugate vaccines (PCV10 and PCV13) compared to no vaccination in Bhutan [36]. It suggested that PCV13 and PCV10 could prevent 30 and 18 deaths respectively in the vaccinated population. If the proportion of the vaccinated population is over 80%, which is likely to be the case in Bhutan, the indirect effect of the vaccine to the unvaccinated population, known as herd protection, could prevent 12 and 10 deaths for PCV13 and PCV10 respectively. Authors concluded that both PCVs would be cost-effective in Bhutan, thus recommending their inclusion in the immunization programme. This has recently been approved by the Bhutanese government, with PCV13 being introduced into the routine vaccination programme in January 2019. The seasonal influenza vaccine is not routinely recommended for at-risk population. Thapa et al. recently published estimations of the number of hospitalizations due to influenza with the aim of informing the Ministry of Health of the seasonal influenza vaccine could be assessed [31]. The benefits of its introduction among recommended target groups are currently being explored by the Bhutanese government.

## Discussion

According to national estimates, ARI and pneumonia are a significant national public health concern in Bhutan. Although pneumonia trends in this country have followed the decreasing trends observed globally, ARI is still responsible for high hospitalisation rates and a significant outpatient burden. Children under five are the most affected group. This is of particular concern, as pneumonia is the single predominant cause of death both in Bhutan and worldwide in this age group. However, there is no robust and comprehensive data in Bhutan describing this illness. Indeed, we did not identify data that would provide a thorough understanding on the aetiology of ARI in Bhutanese children, nor help us characterize this syndrome better. In addition to the scarcity of local data, global estimates might be questionable. Indeed, GBD estimates showed an increase in the LRI mortality rate from 51 to 80 per 100,000 under-five children in 2015 to 100 to 249 in 2016, which is unexpected and doubtful [6, 23]. There were inconsistencies between different local sources with respect to the trend of the burden and mortality of the disease over the years.

Most of the data we identified regarding the aetiology of ARI and pneumonia are from the national ILI and SARI surveillance. This relatively new surveillance system has made great efforts in the last decade, being able to publish weekly epidemiological and virological data from the whole country, through a sentinel surveillance system. Up to now, identification of RSV, HMPV, adenovirus and parainfluenza viruses is attempted only for samples negative for influenza virus. However, targeted screening leads to underreporting of other pathogens, and failure to adequately describe or identify mixed viral infections, commonly occurring in ARI [37, 38]. This issue has been detected and testing all viruses will soon be performed in all the collected samples independently of influenza identification (personal communication). In spite of the moderately good performance of the national surveillance system in place, underreporting appears to be common, questioning therefore the accuracy of the national data provided.

Overall, there is a lack of knowledge regarding the causing pathogens of childhood pneumonia in Bhutan. The national surveillance data showed an increased number of ILI and SARI at the end of the cold season, and during the monsoon. However, no studies were found describing in detail the viral aetiology of ARI in children, in spite of the importance that viruses are known to play in the aetiology of ARI in this age group. Additionally, data on the bacterial aetiology of ARI in Bhutan are also very limited, coming from studies with very low sample sizes [34], from a study on nosocomial VAP, or from limited hospital-based microbiological surveillance efforts. We did not find any study describing other important causative pathogens of respiratory infections in children such as *Mycoplasma pneumoniae*, *Chlamydia pneumoniae*, and *Legionella pneumophila*. We also did not find any study looking at other causes of LRI-like syndrome in children, such as bronchitis, which would fall under the terminology of pneumonia as defined by the WHO. Some of these admissions could be due to non-infectious causes such as hyperreactive airways and contribute to the burden of what is overall classified under the broader umbrella of ARI. Altogether, there are considerable knowledge gaps regarding the aetiology of ARI in Bhutanese children that need to be addressed in a well conducted prospective aetiological study, as a better understanding of the causes of this deadly syndrome, which is crucial for better and more tailored management and preventive strategies. Moreover, comprehensive local data on resistance patterns of causative agents is also needed to effectively design antibiotic management and prevent the emergence and spread of antimicrobial resistance.

Vaccines are possibly one of the greatest public health tools and a well-established strategy to prevent pneumonia and reduce the number of deaths attributed to this illness. Immunization is free of charge for the Bhutanese population. Overall, the country presents high immunization coverage rates, over 95% since 2010, and estimated as 94·4% in 2017 [15]. We found no comprehensive data describing the circulating pneumococcal serotypes in the Bhutanese population, and very limited data describing the burden of pneumococcal diseases in Bhutanese children. Following the recent introduction of PCV13 in the routine immunization programme, it would be highly recommended to monitor the impact of the vaccine. This could be achieved through surveillance system or prospective study to determine circulating pneumococcal serotypes, to estimate the burden of the disease including number of hospitalisations attributed to pneumonia and pneumonia-related mortality, and to assess the interplay between *S. pneumoniae* and viral respiratory infection.

With a small population of less than a million of persons, an elevation ranging from 100 to 7500 m with consequent variety of climate, and a free health care system, Bhutan presents considerable different characteristics compared to the neighbouring countries. Elevation, climate, population density and free health care are all likely to play a role on the epidemiology and aetiology of childhood pneumonia. Therefore, although some data could be extrapolated from neighbouring countries to characterise this disease, we believe that local data are required to fill the knowledge gap on pneumonia in Bhutanese children, given the unique context of this country.

## Conclusions

To our knowledge, this is the first systematic review that gathers evidence from a variety of sources on ARI and pneumonia in Bhutanese children and their significant importance in terms of attributable childhood morbidity and mortality. This is a critical step for summarizing all that is currently known about ARI in Bhutan based on existing data, but also points out the many existing knowledge gaps, which should be addressed so that this syndrome can be better characterised as to its aetiology, epidemiology, patients’ clinico-radiological characteristics and prognostic factors. Data is needed at a national level, as factors that influence the outcomes such as aetiology and patient characteristics vary geographically. Efforts should be made towards the development of research strategies in order to identify causative agents of pneumonia in Bhutan, as well as health determinants and prognostic factors of the illness, so that adequate control measures can be established in the country.

## Supplementary information

**Additional file 1: Appendix 1.** Protocol. **Appendix 2.** Detailed search strategy. **Appendix 3.** List of included records and excluded studies with reasons. **Appendix 4.** Burden of acute respiratory infections in Bhutan. Table 4A. Summary of findings on burden of acute respiratory infections in Bhutan. Table 4B. Incidence and burden of pneumonia in under-five children in Bhutan from 2008 to 2017. Table 4C. Cases of respiratory illness in 2017 and 2018. Table 4D. Incidence of ILI and SARI from 2016 to 2018. **Appendix 5.** Mortality related to acute respiratory infections and pneumonia in Bhutan. Table 5A. Summary of findings on mortality related to acute respiratory infections. Table 5B. Estimates of deaths by cause in children aged 1 to 59 months for Bhutan, from 2000 to 2010. **Appendix 6.** Aetiology of acute respiratory infections. Table 6A. Positivity of samples by age and virus sub-type from 11th June 2009 to 8th August 2010. Table 6B. Pneumococcal serotypes and antibiotic susceptibility from samples collected at JDWNRH in 2014 and 2015. Table 6C. Number of pneumococcal isolates from samples collected at JDWNRH in 2016

## Data Availability

Data sharing is not applicable to this article as no datasets were generated or analysed during the current study.

## Abbreviations

ARI: Acute Respiratory Infections
BHJ: Bhutan Health Journal
GBD: Global Burden of Diseases, Injuries, and Risk Factors Study
Hib: Haemophilus influenza type b
HMPV: Human metapneumovirus
ILI: Influenza-like illness
IMCI: Integrated Management of Childhood Illnesses
JDWNRH: Jigme Dorji Wangchuck National Referral Hospital
KGUMSB: Khesar Gyalpo University of Medical Sciences of Bhutan
LMIC: Low- and middle-income countries
LRI: Lower respiratory infections
PCV10: 10-valent pneumococcal conjugate vaccines
PCV13: 13-valent pneumococcal conjugate vaccines
RCDC: Royal Centre for Disease Control
RSV: Respiratory Syncytial Virus
SARI: Severe Acute Respiratory Infections
UNICEF: United Nations International Children’s Emergency Fund
VAP: Ventilator-Associated Pneumonia
WHO: World Health Organization
