# Supplementary material for: Pneumonia in Bhutanese children: what we know, and what we need to know
Source: Pneumonia (Nathan). 2020 Jan 25;12:1. doi: 10.1186/s41479-019-0065-x (PMC6982390; doi:10.1186/s41479-019-0065-x)
Supplement: Supplementary file 1 — Additional file 1: Appendix 1. Protocol. Appendix 2. Detailed search strategy. Appendix 3. List of included records and excluded studies with reasons. Appendix 4. Burden of acute respiratory infections in Bhutan. Table 4A. Summary of findings on burden of acute respiratory infections in Bhutan. Table 4B. Incidence and burden of pneumonia in under-five children in Bhutan from 2008 to 2017. Table 4C. Cases of respiratory illness in 2017 and 2018. Table 4D. Incidence of ILI and SARI from 2016 to 2018. Appendix 5. Mortality related to acute respiratory infections and pneumonia in Bhutan. Table 5A. Summary of findings on mortality related to acute respiratory infections. Table 5B. Estimates of deaths by cause in children aged 1 to 59 months for Bhutan, from 2000 to 2010. Appendix 6. Aetiology of acute respiratory infections. Table 6A. Positivity of samples by age and virus sub-type from 11th June 2009 to 8th August 2010. Table 6B. Pneumococcal serotypes and antibiotic susceptibility from samples collected at JDWNRH in 2014 and 2015. Table 6C. Number of pneumococcal isolates from samples collected at JDWNRH in 2016 [file 41479_2019_65_MOESM1_ESM.docx]

**Appendices**

**Appendix 1. Protocol**

**Selection criteria**

We will look to include any type of study or any other source that collect data about:

P: children under 5 years of age in Bhutan or all age groups if disaggregated data not available.

I: Acute respiratory infections (ARI) or pneumonia

O: data on:

- burden of ARI such as incidence, prevalence, number of visits in health facilities, and number of hospital admissions; and mortality related to ARI
- aetiology of ARI
- risk factors related to ARI
- clinical description, prognosis and management of ARI
- surveillance systems and national preventive strategies related to ARI

Exclusion criteria: duplication, or when full texts of abstracts not available/found.

**Search methods for identification of studies**

***Information sources***

We will look for potential eligible studies and reported through electronic search of:

- Pubmed, Google Scholar, Science Direct online databases,
- WHO and UNICEF websites,
- Bhutan Health Journal,
- Official reports of Ministry of Health (MoH) in Bhutan, National Statistics Bureau, Royal Centre for Disease Control (RCDC), Bhutan Info, Khesar Gyalpo University of Medical Sciences of Bhutan (KGUMSB) and Jigme Dorji Wangchuck National Referral Hospital (JDWNRH).

We will also look for other sources to identify potential eligible documents, such as:

- Unpublished theses and reports at KGUMSB and JDWNRH,
- Contact with relevant researchers on this area with access to reports not available in the public domain (MoH, JDWNRH, RCDC, etc.)
- Manual check of references of retrieved documents.

***Search query terms***

For Pubmed, we will use the following search strategy: ((respiratory infections[MeSH Terms] OR pneumonia[MeSH Terms]) OR (acute respiratory infection[Title/Abstract] OR pneumonia[Title/Abstract])) AND (Bhutan[MeSH Terms] OR Bhutan[Title/Abstract]). For ScienceDirect and Google scholar, we will use the combination of the following terms: *Acute respiratory infection*, *Pneumonia*, *Pulmonary infection*, *Bhutan and Bhutanese*. We will seek guidance from experts in this field to help improve our established search terms and strategy.

***Language***: no restriction

***Date range***: from available data according to different sources, relevant to the data collected, to the date of the conduct of the search strategy.

**Data collection and analysis**

***Selection of studies***

Two review authors will independently screen the results from our search, from looking at the titles and abstracts, and by checking all the cited sources. We will then retrieve the full-text articles of the potential eligible studies. We will finally assess the full-text articles for eligibility, following our inclusion and exclusion criteria.

***Data extraction and data synthesis***

Two review authors will extract data from the included documents. We will extract data on setting, population, methods and definitions used by the authors. We will collect the outcomes and gather them under different themes:

1. Description of surveillance systems and national preventive strategies in place
2. Burden of ARI
3. Description of the aetiology of ARI
4. Determinants and risk factors of ARI
5. Description of the clinical characteristics and clinical management of ARI

We do not aim to do any metaanalysis of quantitative data. We will summarise our findings in a narrative way, with the support of graphs and tables.

**Appendix 2. Detailed search strategy**

**Pubmed**

| Search | Query |
| --- | --- |
| [#5](https://www.ncbi.nlm.nih.gov/pubmed/advanced) | Search (#3 AND #4) |
| [#4](https://www.ncbi.nlm.nih.gov/pubmed/advanced) | Search (#1 OR #2) |
| [#3](https://www.ncbi.nlm.nih.gov/pubmed/advanced) | Search ((Bhutan[MeSH Terms]) OR Bhutan) OR Bhutanese |
| [#2](https://www.ncbi.nlm.nih.gov/pubmed/advanced) | Search (((acute respiratory infection) OR pneumonia) OR pulmonary infection) OR lower respiratory tract infection |
| [#1](https://www.ncbi.nlm.nih.gov/pubmed/advanced) | Search (respiratory infections[MeSH Terms]) OR pneumonia[MeSH Terms] |

**ScienceDirect**

| Search terms |
| --- |
| Acute respiratory infection AND Bhutan |
| Acute respiratory infection AND Bhutanese |
| Pneumonia AND Bhutan |
| Pneumonia AND Bhutan |
| Pulmonary infection AND Bhutan |
| Pulmonary infection AND Bhutanese |

**Google Scholar**

Bhutan Acute respiratory infection

Bhutan Pneumonia

Bhutan Pulmonary infection

**Appendix 3. List of included records and excluded studies with reasons**

***Included records***

| Studies | Bohler 1995 (1) |
| --- | --- |
|  | Dorji 2018 (2) |
|  | GBD 2018 (3) |
|  | GBD 2017 (4) |
|  | Gupta 2012 (5) |
|  | Tshering 2018 (in press) |
|  | Thapa 2018 (6) |
|  | Tshokey 2017 (7) |
|  | Wangchuk 2013 (8) |
|  | Wangchuk 2011 (9) |
| Reports | Annual Health Bulletins, MoH, from 2009 to 2018 (10) |
|  | Bhutan Health System Review 2017 (11) |
|  | Bhutan Health Situation Trend (12) |
|  | BMIS 2010, UNICEF (13) |
|  | EPI Factsheet Bhutan 2016 (14) |
|  | ILI-SARI Guidelines 2014, MoH (15) |
|  | ILI-SARI Guidelines 2012, MoH (16) |
|  | JDWNRH isolates and susceptibility (unpublished data) |
|  | NEWARS Guidelines 2014 (17) |
|  | RCDC bulletins, weekly Fluview (2 bulletins) (18) |
|  | RCDC bulletins, quarterly (12 bulletins from 2016, 2017 and 2018) (18) |
|  | RCDC monthly epidemiology report (18) |
|  | UNICEF – ARI data per country (19) |

***Excluded studies***

| Study identification | Reason for exclusion |
| --- | --- |
| Caini 2018 (20) | Wrong outcome. Authors looked at the distribution of influenza subtypes within age strata in 29 countries, with no disaggregated data from Bhutan. |
| Caini 2016 (21) | Wrong outcome. This is a retrospective study of epidemiological data, which includes surveillance data from 30 countries. |
| Fisher 2014 (22) | This is a review of the global burden of influenza as a cause of cardiopulmonary morbidity and mortality, including Bhutan. There is no original data, and the few data reported about Bhutan are from studies included in our systematic review (duplicated data). |
| Rutvisuttinunt 2017 (23) | Wrong outcome. Assessment of viral methods in samples negative while tested with conventional methods for influenza. |
| Roth 2015 (24) | Wrong outcome. Systematic review addressing acute respiratory infections case definitions in South Asia. |
| Zhou 2016 (25) | Wrong population. This study analysed retrospectively 121 clinical respiratory samples that were negative by several molecular tests or immunofluorescence assays for identification of viral pathogens with other methods. However, only four of these samples were from Bhutan from 2009 and 2010, the rest of them were from Thailand, Philippines and Nepal. |

**Appendix 4. Burden of acute respiratory infections in Bhutan**

**Table 4A. Summary of findings on burden of acute respiratory infections in Bhutan**

| **Indicators** | **Population** | **Date** | **Main findings** | **Source and ref** |
| --- | --- | --- | --- | --- |
| **Incidence, prevalence** |  |  |  |  |
| Incidence of pneumonia^a^ | <5 years | 2008  2017 | 1479 cases per 10,000 children  809 cases per 10,000 children | MoH, annual health bulletin (10,26) |
| Pneumonia cases^a^ | <5 years | 2008  2017 | 10,626 (5·4% among total morbidity)  5875 (3·7% among total morbidity) | MoH, annual health bulletin (10,26) |
| ARI cases | All ages;  <5 years | 2017 | 171,880 cases  34,835 (20·3%) children | Notifiable disease system (10) |
| ‘Respiratory illness’^b^ | All ages | 2018 | 172,833 cases | RCDC, quarterly bulletins (18) |
| **Hospital visits and inpatients** | | | | |
| Number of inpatients attributed to pneumonia^a^ | <5 years | 2008  2017 | 2554 (31·5% among total hospital inpatients)  1372 (12·3% among total hospital inpatients) | MoH, annual health bulletin (10,26) |
| Outpatient visits attributed to pneumonia in hospitals^a^ | <5 years | 2008  2017 | 3610 (3·3% among total outpatient visits)  1506 (1·8% among total outpatient visits) | MoH, annual health bulletin (10,26) |
| Outpatient visits attributed to pneumonia in BHUs^a^ | <5 years | 2008  2017 | 4462 (5·5% among total outpatient visits)  2924 (4·8% among total outpatient visits) | MoH, annual health bulletin (10,26) |
| Respiratory hospitalization rate | All ages | 2015  2016 | 11,782 cases  13,697 cases | National hospital-based surveillance (6) |
| (discharges coded with respiratory diagnosis) | All ages;  <5 years | 2016 | 3138 respiratory hospitalizations  45% were among children <5 years | Sentinel sites: 6 district hospitals (6) |
| Influenza-associated respiratory hospitalization rates | All ages | 2015  2016 | 50 per 100,000 persons (95% CI 45 to 55)  118 per 100,000 persons (95% CI 110 to 127) | 6 district hospitals identified as sentinel sites (6) |
|  | <5 years | 2015  2016 | 182 per 100,000 persons (95% CI 153 to 210)  532 per 100,000 persons (95% CI 473 to 591) |  |
| Hospital visits and admissions^c^ | Al ages | 2018 | 1214 cases of ILI per 10,000 hospital visits  29 cases of SARI per 100 hospital admissions | RCDC, influenza sen-tinel surveillance (18) |
| VAP | Children between 1 month and 12 years | 2017 | 92 children admitted in the paediatric intensive care unit were included  13 children diagnosed as VAP.  VAP incidence: 14·1%  VAP incidence density: 44·9/1000 ventilator days. | Thesis, JDWNRH (27) |

Abbreviations: ARI: acute respiratory infections; BHUs: basic health units; CI: confidence interval; ILI: influenza-like illness; JDWNRH: Jigme Dorji Wangchuck National Referral Hospital; MoH: Ministry of Health; RCDC: Royal Centre for Disease Control; SARI: severe acute respiratory infections; VAP: ventilator-associated pneumonia.

^a^Detailed data for each year are given in Table 4B.

^b^More details in Table 4C.

^c^More details in Table 4D.

**Table 4B. Incidence and burden of pneumonia in under-five children in Bhutan from 2008 to 2017. Source: Annual Health Bulletins, Ministry of Health** (10)**.**

|  | **Pneumonia incidence**  (per 10,000 CYAR) | **Pneumonia cases**  (% among total morbidity) | **Pneumonia outpatient visits in hospitals**^a^  (% among total outpatient visits in hospitals) | **Pneumonia hospital inpatients**  (% among total hospital inpatients) | **Pneumonia outpatient visits in BHUs**  (% among total outpatient visits in BHUs) |
| --- | --- | --- | --- | --- | --- |
| **2008** | 1479 | 10626 (5·4%) | 3610 (3·3%) | 2554 (31·5%) | 4462 (5·5%) |
| **2009** | 1031 | 7850 (3·9%) | 2863 (2·6%) | 1750 (21·0%) | 3237 (3·8%) |
| **2010** | 1135 | 9204 (5·1%) | 2877 (2·9%) | 2390 (26·2%) | 3937 (5·3%) |
| **2011** | 974 | 7975 (4·7%) | 2642 (2·7%) | 1711 (19·4%) | 3622 (5·7%) |
| **2012** | 1204 | 9939 (5·4%) | 2809 (2·7%) | 2322 (22·2%) | 4807 (7·1%) |
| **2013** | 1080 | 8953 (4·9%) | 2730 (2·6%) | 1451 (17·6%) | 4772 (7·0%) |
| **2014** | 1138 | 9446 (4·8%) | 2924 (2·7%) | 1651 (15·8%) | 4873 (6·5%) |
| **2015** | 905 | 7488 (4·0%) | 1881 (1·8%) | 1537 (12·7%) | 3935 (5·6%) |
| **2016** | 991 | 8134 (4·4%) | 1989 (1·9%) | 2117 (16·3%) | 3909 (6·0%) |
| **2017** | 809 | 5875 (3·7%) | 1506 (1·8%) | 1372 (12·3%) | 2924 (4·8%) |

Abbreviations: BHU: basic health unit. CYAR: children year at risk.

^a^These data exclude the cases from the national referral hospital and from two Indian military hospitals.

**Table 4C. Cases of respiratory illness in 2017 and 2018. Source: RCDC quarterly bulletins** (18)**.**

|  | **2017** | | | | **2018** | | | |
| --- | --- | --- | --- | --- | --- | --- | --- | --- |
|  | Q1 | Q2 | Q3 | Q4 | Q1 | Q2 | Q3 | Q4 |
| ARI (all ages) | 41,022 | 39,809 | 43,536 | 39,878 | 37,655 | 49,376 | 45,273 | 37,845 |
| SARI (all ages) | 548 | 567 | 1021 | 65 | 531 | 988 | 850 | 315 |
| Total respi-ratory illness | 41,570 | 40,376 | 44,557 | 39,943 | 38,186 | 50,364 | 46,123 | 38,160 |
|  | 166,446^a^ | | | | 172,833 | | | |

Abbreviations: ARI: acute respiratory infections; SARI: severe acute respiratory infections

Note: There is a small difference between the 171,880 cases of ARI reported in the annual health bulletin in 2017 with the numbers calculated from the four bulletins available in the RCDC website corresponding to 2017: 166,446 total number of respiratory illnesses and 164,245 cases of ARI.

**Table 4D. Incidence of ILI and SARI from 2016 to 2018. Source: RCDC quarterly bulletins** (18)**.**

|  | **2016** | **2017** | **2018** |
| --- | --- | --- | --- |
| ILI per 10,000 hospital visits (all ages) | Not available | 1610 | 1214 |
| SARI per 100 hospital admissions (all ages) | 32 | 28 | 29 |

Abbreviations: ILI: influenza-like illness; SARI: severe acute respiratory infections.

^a^Data available only for 6 months of the year.

**Appendix 5. Mortality related to acute respiratory infections and pneumonia in Bhutan**

**Table 5A. Summary of findings on mortality related to acute respiratory infections**

| **Indicators** | **Date** | **Main findings** | **Source and ref** |
| --- | --- | --- | --- |
| Under-five deaths | 2015 | 51 to 80 per 100,000 | GBD (3,4)^a^ |
|  | 2016 | 100 to 249 per 100,000 | GBD (3,4)^a^ |
|  | 2016 | 15% attributable to ARI | UNICEF (19) |
| Post-neonatal deaths  (between 1 and 59 months) | 2000^b^  2010 | 230 (28·0%) deaths attributable to pneumonia  120 (27·8%) deaths attributable to pneumonia | Gupta for the MoH (5) |
|  | 2009 to 2011 | 45% attributable to pneumonia | Hospital-based^c^ (5) |
|  | 2016 | 27% attributable to ARI | UNICEF (19) |
| Children between 1months and 12 years | 2018 | 5 deaths over a year among children with VAP | JDWNRH (27) |
| All population | 2017 | 5^th^ main cause of death (‘pneumonia’) | MoH, annual health bulletin (10) |

Abbreviations: GBD: Global Burden of Diseases, Injuries, and Risk Factors Study; MoH: Ministry of Health; VAP: ventilator-associated pneumonia.

^a^The GBD study provides data on the burden of LRI in 195 countries, including Bhutan. The GBD team used literature review and country-level covariates to produce modelled estimates such as LRI mortality, LRI incidence, hospital admissions due to LRI, risk factors for LRI mortality and LRI burden attributed to four high-burden aetiologies. However, disaggregated data per country are not available for all these estimates.

^b^Detailed data for each year from 2000 to 2010 are given in Table 5B.

^c^Data on child mortality happening in health facilities (at the national referral hospital, the two regional referral hospitals and seven of the district hospitals), which is likely to overestimate the true value.

**Table 5B. Estimates of deaths by cause in children aged 1 to 59 months for Bhutan, from 2000 to 2010. Source: Gupta for the Ministry of Health in Bhutan** (5)**.**


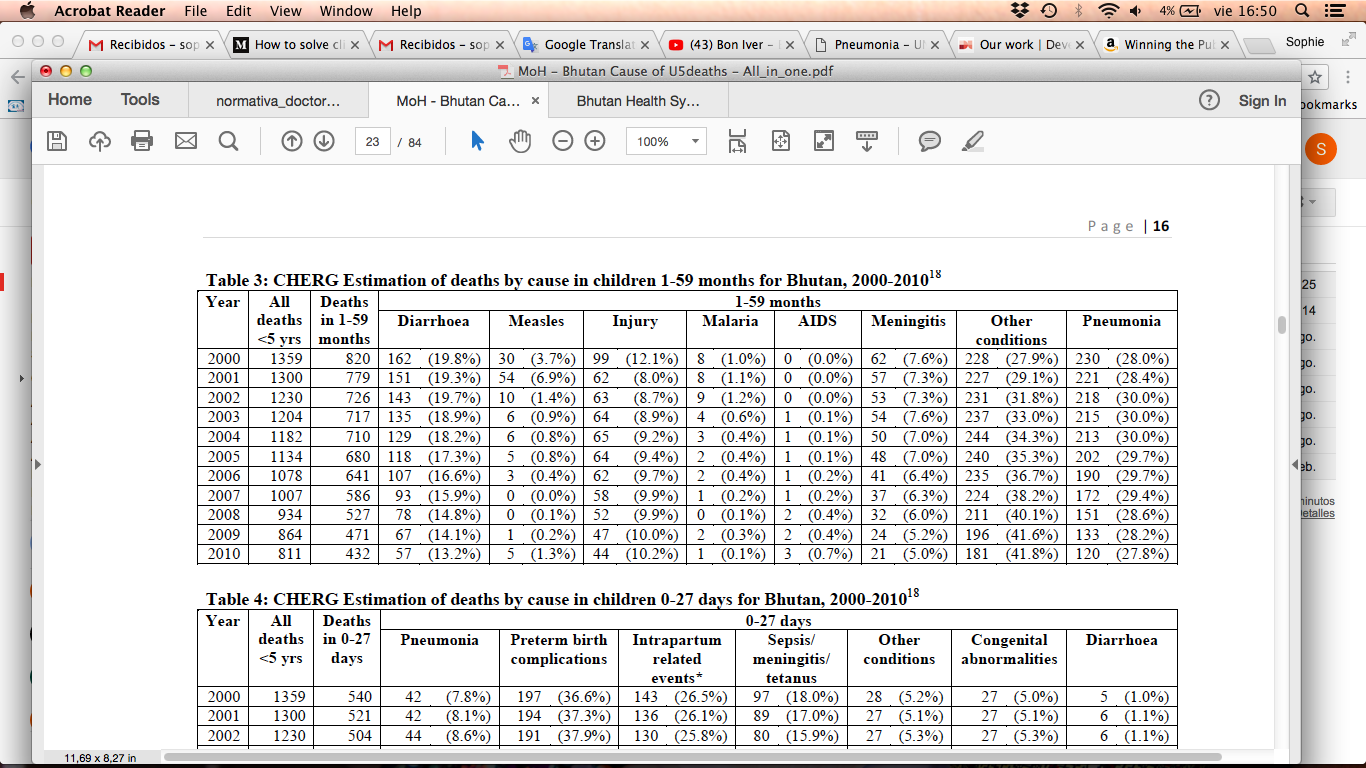


**Appendix 6. Aetiology of acute respiratory infections**

**Table 6A. Positivity of samples by age and virus sub-type from 11^th^ June 2009 to 8^th^ August 2010. Reproduction permitted. Source: Wangchuk 2013** (8)**.**


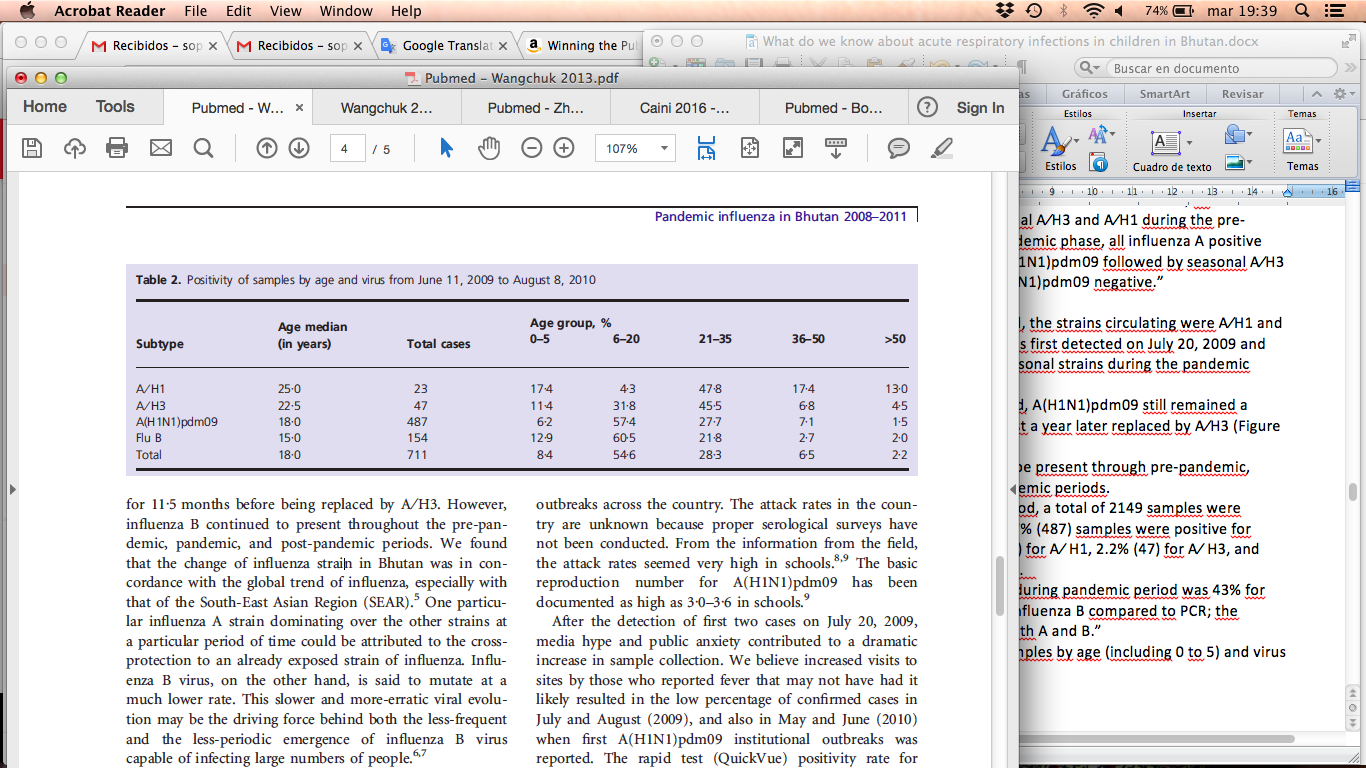


**Table 6B. Pneumococcal serotypes and antibiotic susceptibility from samples collected at JDWNRH in 2014 and 2015. Source: Tshokey et al** (7)**.**

| Type of sample | Eye secretions (10)  Blood (5)  Throat frotis (2)  Sputum (2)  Ascitic fluid (1)  Pus (1) |
| --- | --- |
| Serotypes identified^a^ | Three 10A  Two 1, 6B and 19F  One each of 4, 6A, 6C, 7C, 7F, 9V, 15B, 19A, 33C, 38, 41 and non-typeable |
| Antibiotic susceptibility | All isolates sensitive to penicillin, chloramphenicol, and ceftriaxone  9.5% of isolates resistant to erythromycin, and 38.1% to cotrimoxazole. |

^a^Although 37 isolates were first identified as *S. pneumoniae*, only 21 were confirmed to be *S. pneumoniae* and were serotyped.

**Table 6C. Number of pneumococcal isolates from samples collected at JDWNRH in 2016. Source: personal communication, unpublished.**

| **Type of sample** | **Number of isolates** | **Antibiotic susceptibility (%)** | | | |
| --- | --- | --- | --- | --- | --- |
|  |  | Penicillin | Erythromycin | Cotrimoxazole | Chloramphenicol |
| Blood | 9 | 100 | 89 | 17 | 88 |
| Respiratory | 12 | 100 | 83 | - | - |

**References**

1. Bøhler E, Aalen O, Bergstram S, Halvorsen S. Breast feeding and seasonal determinants of child growth in weight in East Bhutan. Acta Pædiatrica. 1995;84(9):1029–34.

2. Dorji K, Phuntsho S, Pempa, Kumluang S, Khuntha S, Kulpeng W, et al. Towards the introduction of pneumococcal conjugate vaccines in Bhutan: A cost-utility analysis to determine the optimal policy option. Vaccine. 2018;36(13):1757–65.

3. Troeger C, Blacker B, Khalil IA, Rao PC, Cao J, Zimsen SRM, et al. Estimates of the global, regional, and national morbidity, mortality, and aetiologies of lower respiratory infections in 195 countries, 1990–2016: a systematic analysis for the Global Burden of Disease Study 2016. Lancet Infect Dis. 2018;18(11):1191–210.

4. Troeger C, Forouzanfar M, Rao PC, Khalil I, Brown A, Swartz S, et al. Estimates of the global, regional, and national morbidity, mortality, and aetiologies of lower respiratory tract infections in 195 countries : a systematic analysis for the Global Burden of Disease Study 2015. Lancet Infect Dis. 2017;17:1133–61.

5. Gupta S. Identification of causes of under-five deaths in health facilities in Bhutan. 2012.

6. Thapa B, Roguski K, Azziz-Baumgartner E, Siener K, Gould P, Jamtsho T, et al. The burden of influenza-associated respiratory hospitalizations in Bhutan, 2015-2016. Influenza Other Respi Viruses. 2018;(March):1–8.

7. Tshokey T, Yangzom D, Sharma R, Tshering N. Serotypes and antibiotic susceptibility of Streptococcus pneumoniae in the Jigme Dorji Wangchuck National Referral Hospital, Thimphu, Bhutan: a preliminary finding. Bhutan Heal J [Internet]. 2017;3(1):13–8. Available from: http://www.bhj.com.bt/index.php/bhj/article/view/42

8. Wangchuk S, Thapa B, Zangmo S, Jarman RG, Bhoomiboonchoo P, Gibbons R V. Influenza surveillance from November 2008 to 2011; including pandemic influenza A(H1N1)pdm09 in Bhutan. Influenza Other Respi Viruses. 2013;7(3):426–30.

9. Wangchuk S, Zongmo S, Thapa B. Epidemiological analysis of Influenza – Like Illness and Severe Acute Respiratory Infection surveillance for 2011. 2011;

10. Ministry of Health. Annual Health Bulletin [Internet]. Thimphu; 2018 [cited 2019 Jan 26]. Available from: http://www.health.gov.bt/publications/annual-health-bulletins/

11. WHO. The Kingdom of Bhutan Health System Review. Vol. 7, Health Systems in Transition. 2017.

12. WHO. Bhutan Health Situation Trend [Internet]. Available from: http://www.searo.who.int/entity/health_situation_trends/data/hsp/bhutan_hsp.pdf

13. National Statistics Bureau. Bhutan Multiple Indicator Survey [Internet]. 2010 [cited 2019 Sep 11]. p. 1–327. Available from: http://www.nsb.gov.bt/main/main.php

14. WHO. EPI Fact Sheet Bhutan [Internet]. 2016 [cited 2019 Sep 10]. Available from: http://www.searo.who.int/immunization/data/bhutan.pdf

15. Ministry of Health Bhutan. Operational Guideline for Influenza-Like Illness and Severe Acute Respiratory Infection. Thimphu; 2014.

16. Ministry of Health Bhutan. Operational guideline for ARI, ILI & SARI Surveillance. Thimphu; 2012.

17. Public Health Laboratory. National Notifiable Disease Surveillance System and Epidemiology Unit. Thimphu; 2014.

18. Ministry of Health Bhutan. Royal Centre for Disease Control [Internet]. 2018 [cited 2019 Jan 16]. Available from: http://www.rcdc.gov.bt/

19. UNICEF. Estimates of child cause of death, Acute Respiratory Infection. 2018.

20. Caini S, Spreeuwenberg P, Kusznierz GF, Rudi JM, Owen R, Pennington K, et al. Distribution of influenza virus types by age using case-based global surveillance data from twenty-nine countries , 1999-2014. BMC Infect Dis. 2018;18:269–78.

21. Caini S, Andrade W, Badur S, Balmaseda A, Barakat A, Bruno A, et al. Temporal Patterns of Influenza A and B in Tropical and Temperate Countries: What Are the Lessons for Influenza Vaccination? PLoS One [Internet]. 2016;11(3):1–15. Available from: http://dx.doi.org/10.1371/journal.pone.0152310

22. Fischer WA, Gong M, Bhagwanjee S, Sevransky J. Global Burden of Influenza as a Cause of Cardiopulmonary Morbidity and Mortality. Glob Heart [Internet]. 2014;9(3):325–36. Available from: http://dx.doi.org/10.1016/j.gheart.2014.08.004

23. Rutvisuttinunt W, Klungthong C, Thaisomboonsuk B, Phonpakobsin T, Lon C, Saunders D, et al. Retrospective use of next-generation sequencing reveals the presence of Enteroviruses in acute in fluenza-like illness respiratory samples collected in South / South-East Asia during 2010–2013. J Clin Virol [Internet]. 2017;94(2017):91–9. Available from: http://dx.doi.org/10.1016/j.jcv.2017.07.004

24. Roth DE, Gaffey MF, Smith-romero E, Fitzpatrick T, Morris SK. Acute respiratory infection case definitions for young children: a systematic review of community-based epidemiologic studies in South Asia. Trop Med Int Heal. 2015;20(12):1607–20.

25. Zhou Y, Fernandez S, Yoon I, Simasathien S, Watanaveeradej V, Yang Y, et al. Metagenomics Study of Viral Pathogens in Undiagnosed Respiratory Specimens and Identification of Human Enteroviruses at a Thailand Hospital. Am J Trop Med Hyg. 2016;95(3):663–9.

26. WHO. Bhutan Health situation trends [Internet]. 2013 [cited 2016 Oct 6]. Available from: http://www.searo.who.int/entity/health_situation_trends/data/hsp/bhutan_hsp.pdf

27. Tshering T. Incidence, risk factors, and outcome Of Ventilator-Associated Pneumonia in Pediatric intensive care unit Jigme Dorji Wangchuk National Referral Hospital. A prospective hospital-based study. KGUMSB; 2018.
